# Epistemic artifacts and the modal dimension of modeling

**DOI:** 10.1007/s13194-021-00374-5

**Published:** 2021-07-12

**Authors:** Tarja Knuuttila

**Affiliations:** Department of Philosophy, University of Vienna, Universitätsstraße 7, 1010 Wien, Austria

**Keywords:** Models, Epistemic artifacts, Modalities, Representation, Economic modeling, Synthetic biology

## Abstract

The epistemic value of models has traditionally been approached from a representational perspective. This paper argues that the artifactual approach evades the problem of accounting for representation and better accommodates the modal dimension of modeling. From an artifactual perspective, models are viewed as erotetic vehicles constrained by their construction and available representational tools. The modal dimension of modeling is approached through two case studies. The first portrays mathematical modeling in economics, while the other discusses the modeling practice of synthetic biology, which exploits and combines models in various modes and media. Neither model intends to represent any actual target system. Rather, they are constructed to study possible mechanisms through the construction of a model system with built-in dependencies.

## Introduction

The philosophical discussion of models has had many beginnings, generating diverse analyses and targeting different kinds of models. There is one common theme, however, that ties much of this seeming heterogeneity together: representation. Philosophers of different orientations and philosophical commitments have considered, despite their diverging views, models as representational vehicles or even defined them on the basis of their representational function. (e.g. Chakravartty, 2010; Giere, 2010; Teller, 2001). Yet, conceiving of models as representations has not led to any noticeable consensus, the notion of representation itself engendering an assorted literature of its own. In this paper, I further develop a novel candidate for a unified approach, that of approaching models as epistemic artifacts. Rather than assuming, at the outset, that models are representational entities, and consequently attributing their epistemic value to representation, the artifactual approach focuses on model construction, which is key to understanding how a model can achieve its epistemic purposes.

I will argue that since the unit of analysis of the artifactual approach is not the representational model-target pair, it is more apt for dealing with modal dimension of modeling, and in particular those models whose targets are either non-actual, hypothetical or generic. The artifactual account has been outlined by Knuuttila (2005, 2011) and discussed so far in relation to engineering sciences (Currie, 2017; Knuuttila & Boon, 2011), fiction (Currie, 2017; Knuuttila, 2017), and idealization (Carrillo & Knuuttila, forthcoming). As the idea that models give us knowledge by virtue of representation is so well established in the philosophy of science community, I will use two brief case studies to exemplify how one can explain the epistemic value of models without assuming that they represent actual target systems. The first case portrays mathematical modeling in economics, while the other discusses the modeling practice of synthetic biology that exploits and combines models in various materialities. In particular, I will focus on how, in each case, models were carefully constructed to study possible mechanisms^1^ through the construction of a model system with built-in dependencies.

## The representational approach

The artifactual approach analyzes scientific models as purposefully designed human-made or human-altered objects that are used in view of particular questions or aims in the context of specific scientific practices (Knuuttila, 2011, 2017). This approach aims to provide an alternative to the representational account of models, seeking to avoid the problems of the latter when it comes to understanding the epistemic functioning of modeling. The two approaches, the representational and the artifactual, do not necessarily clash, depending on how the notion of representation is analyzed. The point is, however, that in order to understand how scientists gain knowledge by modeling, the supposed representational relationship between a model and a target system is not the best place to begin, but rather the construction of particular kinds of epistemic objects, models, and the characteristic ways they are used in science. Representation plays multiple roles in this process, of which some, rather paradoxically, are disregarded by the representational approach to modeling. (I will return to this topic in the next section.)

To understand why the artifactual approach offers a better account of model-based representation, let us start from the epistemological picture that motivates the *traditional* representational approach. This picture merges the representationalist theory of knowledge with metaphysical realism. According to metaphysical realism, the world that science investigates and scientific theories describe, is mind-independent.^2^ Representationalist theory of knowledge, in turn, conceives of knowledge as a collection of mental or external representations that reproduce accurately, or stand truthfully for, parts of this mind-independent world. The central problem for such representationalist theory of knowledge is to explain how representations are supposed to stand for something else. In other words, what is it that provides this access to the mind-independent and hence representation-independent world? One solution to this problem is to invoke a privileged layer in representations that would ground knowledge. Philosophy and cognitive sciences are replete with proposals of this kind: immediate ideas, concepts, logical forms, and so on. Structures have played this role in philosophy of science. Thus, in discussing various kinds of material models, French and Ladyman affirm that “the specific material of the model is irrelevant; rather it is the structural representation, in two or three dimensions, which is all important.” (1999, 109).

The unit of analysis of the (traditional) representational account of models – the model-target dyad – reflects the aforementioned epistemological portrayal. According to it, a model is considered as a representation of a worldly target system, and it gives knowledge of the target if it succeeds to accurately depict at least some aspects of this target system. The structuralist approach exemplifies the representational approach in its purest form; it attributes representation to a structure that is shared by both the model and its target system (e.g. French, 2003; French & Ladyman, 1999; Pincock, 2004). Apart from offering a clear-cut account of the representational relation, the structuralist approach is also appealing in that it simultaneously provides a criterion for the representational success. However, the pragmatists of scientific representation have singled out many shortcomings of the structuralist account (Frigg, 2006; Suárez, 2003), arguing that the unit of analysis should be expanded to cover also the users, the aims and norms of representation (Giere, 2010; Suárez, 2004), and even other pragmatic features such as audience (Mäki, 2011). This extension of the unit of analysis comes with a price. Due to the deflationary nature of pragmatic accounts, they do not possess resources to explain the epistemic value of modeling. Not that they would attempt to do this either, the deflationary nature of pragmatic accounts is an intended feature of theirs, as Suárez and Pero (2019) explain:

Deflationists do not require an explanation of the capacity of a scientific model source to license inferences about its target—particularly not so in virtue of any deeper features of the representational relation between source and target. (354-355)

Not all pragmatists have been this deflationary. Frigg and Nguyen have recently developed an elaborate pragmatic account of representation that applies to it the notions of exemplification and make-believe (e.g. Frigg & Nguyen, 2016, 2020). On the other hand, the structuralists have added pragmatic elements to their version of the inferentialist account of representation (e.g. Bueno & Colyvan, 2011; Bueno & French, 2011). While Bueno and French acknowledge the importance of target construction, and especially the role of mathematics in it, they (still) approach representation in terms of structural mappings between the model and the target system (e.g. Bueno & French, 2018).

Three takeaways can be drawn from the present philosophical discussion of representation:

(i) Representation can be approached in nearly opposite ways: the unit of analysis is not shared by the structuralists and the pragmatists.
(ii) While no consensus has emerged, the structuralists have cautiously accepted the idea of target construction thus adding pragmatic elements to their analyses.
(iii) Pragmatist approaches tend to be deflationary in that they do not explain how and why models give us knowledge.

Given this multiplicity of philosophical accounts of representation, any claim that models give knowledge of their targets systems by virtue of representing them should be accompanied by an explication of what notion of representation is being referred to. Moreover, if one subscribes to some pragmatic account of representation, one should seek an answer to the epistemic value of models from the particular modeling practices themselves, due to the deflationary nature of these accounts. Finally, the recent discussion of modeling has also sought to loosen the representational grip of the model-target dyad, by focusing, instead of representation, on how scientists learn from models by constructing and manipulating them (Morrison & Morgan, 1999), or considering them independent from any single, uniquely determinable relationship to the world (Weisberg, 2007, 218). Consequently, even though the idea of representation seems intuitively appealing, and even self-evident, attributing the epistemic value of modeling to representation is not doing too much philosophical work in and of itself. Given the too stringent requirements of structuralism, and the deflationary nature of pragmatism, the artifactual approach makes a fresh start in not tying the epistemic value of modeling to the supposed representational relationship between the model and some worldly target system.

## Models as epistemic artifacts

The artifactual approach focuses on how conceptual, theoretical, mathematical or computational *access* to some system of interest is gained through the employment of diverse epistemic tools. Apart from evading the difficulties concerning representation, the artifactual approach to modeling has other important benefits. First, it is able to accommodate many modeling practices that seem epistemically inferior according to representationalist criteria. For example, abstract, highly idealized models have been subject to longstanding criticism in both the biological sciences and economics. The artifactual approach is able to provide a more flexible account of the epistemic advantages of modeling in those fields. Second, a big part of the modeling endeavor across the sciences investigates various possibilities, involving not only actual entities and processes, but also non-actual ones. The representational model-target dyad does not seem to furnish an adequate unit of analysis for models without any actual determinable targets.

The artifactual approach to modeling stands on two pillars: (i) the constrained construction of a model that is due to its intended use(s), and (ii) the representational tools used in producing a model.^3^ In this section, I will discuss both of them, proceeding then to consider the question of justification as well as the modal dimension of modeling.

### Constrained construction

From the artifactual perspective, models are particular kinds of epistemic artifacts that function as *erotetic devices.* That is, they are artificial systems of dependencies that are constrained in view of answering a pending scientific question, motivated by theoretical and/or empirical considerations. Typically, a lot of theoretical and empirical knowledge is already built into the model, both in terms of its specific construction and the question(s) it is designed to investigate. Much of this knowledge is implicit, and can only be appreciated by competent users in the context of particular scientific practices. Given this built-in knowledge as well as the erotetic function of models, the problem of representation is not as acute as it seems: models are not isolated entities in need of connection to worldly systems by a relation of representation. The epistemological puzzle of how the representational relationship between a model and the world should be analyzed becomes that of studying how the model construction facilitates the study of pending scientific questions.

The notion of a constraint is crucial for such facilitation: in order to achieve its epistemic aims the model needs to be properly limited. The philosophical discussion of idealization has paid attention to the constrained nature of models, highlighting how models are designed to isolate some supposedly relevant features of the system of interest, disregarding and/or distorting the rest (Cartwright, 1999; Mäki, 1992; Strevens, 2008). On the other hand, other philosophers have emphasized that the use of mathematical and statistical methods entails distortion, often with considerable epistemic benefits (McMullin, 1985; Rice, 2018). While in the discussion of idealization, philosophers have tended to examine either the benefits or shortcomings of idealization, from the artifactual perspective, idealizations typically are epistemically both beneficial and detrimental (Carrillo & Knuuttila, forthcoming). They enable certain questions to be inquired while overlooking others. This epistemic two-sidedness does not just concern idealizations, but the use of representational tools more generally.

Consequently, in contrast to the representational approach that zeroes in on the relationship of representation, the artifactual approach attends to the enablings and limitations of the representational tools that scientists employ in model construction. It recognizes that in order for a model to provide access to the actual or possible aspects of the world, it needs to have a sensuously available workable dimension rendered by various representational tools. In highlighting such a concrete dimension of models, the artifactual approach follows Morrison and Morgan (1999), who called attention to how scientists *learn* by building and manipulating models. This constructive and interventional side of modeling is crucial for the epistemic value of models: scientists gain knowledge through investigating and articulating different relationships built into the model. There is no need to suppose that the only route to knowledge is through (at least) partially accurate reproduction of the actual state of affairs in the world. In contrast, models as artifacts allow epistemic access to many theoretical and empirical problems by enabling various inferences (Suárez, 2004), providing novel results, prompting new experiments – and, in so doing, they possess considerable modal reach (Godfrey-Smith, 2006).

### Representational tools and internal vs. external representation

The representational tools used in model construction can be analyzed according to the modes and media they embody. The *representational mode* refers to the different symbolic or semiotic devices (pictorial, linguistic, mathematical, diagrammatic etc.) with which various meanings or contents can be expressed. Such representational modes are embedded in *representational media* that encompasses the material means with which representations are produced (such as ink on paper, a digital computer, biological substrata and so forth). For instance, natural language is a representational mode that can be realized by different media, either as speech or as writing (Knuuttila, 2011).^4^ Materiality may play different epistemic roles depending on the type of a model in question, as shown below by the synthetic biology case. The synthetic repressilator and the electronic repressilator both instantiate the same ring oscillator design, i.e. the same representational mode, yet they are implemented in different media, enabling different kinds of inferences.

It may sound contradictory to claim that the artifactual approach tries not to invoke representation, yet addressing, at the same time, representational tools as central for the epistemic access to the world. No such contradiction is involved, however. In order to see this, two different notions of representation need to be distinguished: internal and external.

Internal representation has to do with how various kinds of sign-vehicles or representational devices are used to make meaning and convey content.^5^ In talking about ‘content’ I do not want to imply that any sign-vehicle, or representational device, would carry or correspond to a stable unit of content; meaning-making always depends on the linguistic and other practices in place, as well as the context of use. By external representation, I refer to the relationship of a model to a real-world target system, the question on which the philosophical discussion has largely concentrated. To use a toy example of the differences between these two notions of representation, let us consider traffic signs. The meaning of a Dangerous Turn road sign can be understood internally in relation to the system of traffic signs (the system itself being grounded on automobile traffic, the practices of regulating it, possible forms of roads, etc.). The sign has the function of warning of the dangerous turn only when placed in an appropriate place, in which case it can be taken as an external representation of a nearby dangerous turn, whose intended use is to caution drivers of it. But, if detached from such a context, its meaning would still be understood by people knowledgeable of the system of traffic signs.

Philosophy of science has not explicitly distinguished between external and internal representation—with the exception of the discussion of fiction.^6^ One may wonder whether philosophers’ strong conviction that modeling has something to do with representation is at least partially due to such conflation. Nevertheless, the fact that something may be internally represented within a model without necessarily representing the actual state of worldly affairs opens up the prospect of conceiving modeling as a practice of exploring the possible.^7^

### Distributed justification

A proponent of the representational approach might be fine with all of the aforementioned aspects of the artifactual approach, but may still urge the artifactualist to account for the various inferences, new results and learning derived from models. After all, the representational relation is also expected to provide justification for model-based results – except for the deflationary pragmatists, who separate the analysis of the notion of representation from its success. The traditional representational story is neatly packaged, but comes too cheaply. From the artifactual perspective, any (external) representational relation a model might bear to some worldly target is always an accomplishment to be independently justified – instead of being a privileged relation providing a warrant for the truth or correctness of model-based results. The justification of models, and the interpretations based on them, is two-fold. It is partly built-in due to the already established theoretical, empirical and representational resources called upon and utilized in model construction (Boumans, 1999), and partly a result of the triangulation of different epistemic means: other models, experiments, observations and background theories. Such processes of triangulation are distributed in terms of epistemic labor, likely very complex and indirect, and usually inconclusive in character.

### The artifactual account and the modal dimension of modeling

That modeling involves the study of different possibilities, and often only presents how-possibly explanations, has been recognized by many philosophers of science. However, only relatively recently has there been a more concerted effort to understand the modal nature of modeling. For any progress in accounting for how models enable the study of possibilities, one would first need to know what kind of possibility is at stake. Often when modelers espouse possibility claims, their function may only be apologetic, expressing the scientists’ lacking or only partial commitment to the underlying assumptions or results of the model (Gruene-Yanoff, 2019). Another trivial use of the notion of possibility is that of considering how-possibly models as incomplete how-actually models (e.g. Craver & Darden, 2013). From the modal perspective, the distinction between epistemic possibility and objective possibility seems more far-reaching (Sjölin Wirling & Gruene-Yanoff, 2021). Epistemic possibility, in the scientific context, depends on scientific knowledge of the actual state of the world. Any scientific claim that is not ruled out by scientific knowledge is epistemically possible. Consequently, there can be several epistemically possible models of a certain actual target system. In contrast, objective possibility can also concern some unactualized state of the world. (Verrault-Julien & Gruene-Yanoff, forthcoming). As a result, there can be (epistemically) possible models of actual targets and models of (objectively) possible targets^8^ (Gruene-Yanoff, 2019). Tackling the latter kinds of models seems more challenging from the representational perspective than from the artifactual perspective, since the target system is nonexistent, and only possible.

What are models then addressing, if not actual target systems? In discussing what she calls modal understanding, Le Bihan (2016) notices how the understanding of phenomena and the understanding of the actual world are often conflated. Her account of modal understanding addresses phenomena instead of the actual world, and it does so in terms of dependency structures that enable scientists “to navigate the possibility space” (122). With such “navigation” she refers to knowledge of (i) how a particular dependency structure gives rise to (some subset of) a phenomenon (ii), how the dependency structures are related to each other, and (iii), “how some general constraints apply to the entirety of the possibility space.” (118) The increasingly popular talk of “possibility spaces” is often only hypothetical, bound to a synchronic view of possibilities, and does not apply to all modal reasoning. That said, Le Bihan’s idea of modal understanding being gained from the knowledge of how different dependency structures are able to engender certain kinds of phenomena is enlightening. Such modal understanding fits the artifactual approach, which approaches models as purposefully constructed systems of interdependencies designed to answer some pending scientific questions. These scientific questions typically inquire into how certain phenomena could be generated or maintained, or how robust it is.

Obviously, the question then becomes that of whether the dependency structures studied through modeling really do capture some objective possibilities. Massimi (2019) has suggested that “physical conceivability”, either law-bounded or law-driven, could be crucial for such inferences. While invoking law-boundedness, or drivenness, does not suit many other disciplines in the same way as it does physics, the question of conceivability is crucial for modal inferences nevertheless – as the following two case studies will show. Both the economic and biological models discussed below lay out an artificial system of interdependencies able to generate certain phenomena of interest. It is difficult to point out any actual target systems for either of these models, as they instead appear to address objective possibilities.

## Imaginary economies

During the 1950s and 1960s, the Monetarists, with Milton Friedman leading the charge, challenged the reigning Keynesian macroeconomics. Friedman’s perhaps most famous claim concerned the causation of inflation. He claimed that “inflation is always and everywhere a monetary phenomenon […] in the sense that it is and can be produced only by a more rapid increase in the quantity of money than in output.” (Friedman, 1970b, 24) Friedman and his co-workers presented different kinds of empirical evidence for the causal priority of money for nominal income and inflation. The most important evidence concerned the timing of leads and lags of money supply and nominal income. The cyclic turning points in money income were found to be preceded by turning points in the rate of change in money supply M’ and money stock M (e.g., Friedman, 1961). These leads and lags seemed to imply that causation runs *from* money supply *to* nominal income and inflation.

Friedman’s critics were quick to point out that a correlation between the changes of money supply and income does not necessarily entail a causal relation between the two, and that there are well-known philosophical arguments against inferring causal priority from temporal priority (Brainard & Tobin, 1968; Clark, 1960; Culbertson, 1960). Furthermore, the timing sequences found by Friedman and his co-workers also were questioned for possibly being a mathematical artifact (e.g., Clark, 1960; Culbertson, 1960; Sims, 1992). Tobin (1970a) entered this discussion with an entirely different strategy, that of modeling. His article “Money and Income: Post Hoc Ergo Propter Hoc” (1970a) has become a classic in monetary economics.

The name of the article already conveys Tobin’s basic message. Post hoc ergo propter hoc (after this, therefore because of this) refers to the aforementioned fallacy of inferring causal priority from the order of events. Tobin’s strategy is to take Friedman’s empirical claims concerning leads and lags at face value and ask whether it is possible that such temporal findings could be produced by an entirely different causal mechanism than the one advocated by the Monetarists. In order to study this question Tobin constructed a model to demonstrate that Friedman’s findings on timing do not show that “changes in the supply of money M […] are the principal cause of changes in the money income Y” (Tobin, 1970a, 301). In fact, Tobin constructs two models, an “ultra-Keynesian” one and a “Friedmanian” one. In these two models, money has totally opposite causal roles. In the ultra-Keynesian model, autonomous investment and government expenditure determine income to which money supply responds passively. In contrast, in the Friedman model the money supply and its rate of change are autonomous, determined by monetary authorities. The ingenuity of Tobin’s modeling exercise is to show that the ultra-Keynesian model can generate the timing sequences of Friedman’s empirical studies. In other words, even though the changes in the rate of money supply and monetary stock precede the changes in nominal income, Tobin’s model shows that they “can be generated by a structure in which money has no causal role” (Tobin, 1970b, 329).

The representational account of models does not capture Tobin’s accomplishment, as he makes no claims concerning actual economies. His model amounts to a rather basic exercise in Keynesian model-building, being a toy model at best. For instance, investment in the model is insensitive to the interest rate, and the price level is fixed. The money supply is endogenous, it adapts passively to the needs of trade. Tobin himself is very clear about the imaginary nature of his model and warns against taking it as a representation of any real economy: “I hasten to say that I do not believe the ultra-Keynesian model to be exhibited” (1970a, 303). The ultra-Keynesian model is a purposefully built construction that does not seek to be too faithful to Keynesian theory either. Friedman’s and Tobin’s subsequent exchange is interesting in this regard. Friedman claims that the demand for money equation in the ultra-Keynesian models is actually not genuinely Keynesian (Friedman, 1970a). Tobin responds that the money demand equation should not be taken by itself, but related to the “model as a whole” (Tobin, 1970b, 329).

Tobin mentions that part of the popular and semi-professional appeal of Friedman’s brand of monetarism derives from his and his co-workers research on the *timing* of economic aggregates. Consequently, with the ultra-Keynesian model, Tobin seeks to show that temporal lead in monetary aggregates does not imply their causal priority. Tobin writes: “*Every single piece of observed evidence that Friedman reports on timing is consistent with the timing implications of the ultra-Keynesian model* […]” (1970a, 315, italics in the original). And yet, in the ultra-Keynesian model, money is totally dependent on the determinants of nominal income.

In sum, the ultra-Keynesian model provides an example of the modal dimension of modeling: it establishes an economic possibility. In terms of Le Bihan’s distinction between phenomena and the world, the ultra-Keynesian model addresses merely economic phenomena, as Tobin is not committed to the model representing any actual economic system. Instead, he constructs two artificial model economies to study the question of whether the phenomenon of leads and lags found by Friedman could be accounted for by radically different dependency relations between economic aggregates. It is the modal understanding brought about by Tobin’s ultra-Keynesian and Friedmanian models that explains the continued acclaim of Tobin’s article, not merely the demonstration that temporal priority does not establish causal priority.

Of course, the question remains whether such an economic system is possible, in which money supply is entirely dependent on the determinants of income. Tobin is silent on whether an economic system even somewhat like the ultra-Keynesian one *could* exist. Answering this question would require the consideration of its *economic conceivability*. There are no economic laws that would ground such conceivability, but various theories of endogenous money supply have been advanced from Knut Wicksell and Joseph Schumpeter in the nineteenth century to Kaldor (1982) and contemporary Post-Keynesian Economics. Central banks unquestionably can and do influence money supply, but does this need to be the case? Elsewhere, in other disciplines, scientists have sought to study whether the possible systems studied by modeling could be actualized. Synthetic biology provides a prime example of such an endeavor.

## Biological oscillators in various modes and media

Synthetic biology is mainly known by scientific audiences and the public alike by way of its ambitious engineering aims. Yet, alongside its engineering agenda, synthetic biology has pursued more general theoretical questions. As Nature put it 2010 in its editorial in an issue on synthetic and systems biology: “the contributions to and from basic science are the part of synthetic biology that most deserves celebration” (Nature, 2010). What Nature is referring to as the “basic science part of synthetic biology”, is the aim of synthetic biology to design and define minimal biological systems to implement a particular function. In this section, I will concentrate on the repressilator^9^ and the research program of synthetic oscillators that it spawned. These oscillators do not seek to recreate any existing circuits, but are rather constructed to probe possible biologically viable circuit designs. As such, this research program, and synthetic biology more generally, is geared towards examining potential biology (Elowitz & Lim, 2010). The research on synthetic oscillators triangulates mathematical models with simulations and synthetic biological systems, including even electronic analogues of synthetic systems. It provides an interesting case in regards to the various representational and other artifactual vehicles used in modeling.

### Mathematical, synthetic and electronic repressilators

Living organisms keep track of time, and such periodic rhythms take place also on the intracellular level; daily rhythms have been observed even in single-celled organisms like cyanobacteria (Bell-Pedersen et al., 2005). The first ideas of how they are able to perform such rhythmic behavior patterns emerged in the early 1960s, when researchers began to model genetic and metabolic regulation in terms of feedback systems, where e.g. genes are controlled at the level of transcription by the products of other genes. Various mathematical models and simulations of different kinds of biological oscillators have been presented since, and circadian clock genes have been experimentally identified in different model organisms. Yet, despite this theoretical and experimental work, there was no direct confirmation of the biological realizability of such intracellular feedback mechanisms – introduced to biology from engineering and physics – until the onset of synthetically created systems. The repressilator (Elowitz & Leibler, 2000) provided proof of concept.

The repressilator is a synthetic circuit that consists of three repressor proteins, which are configured in such a way that they repress the protein production of their neighbor gene in the fashion of the game rock-paper-scissors (Gao & Elowitz, 2016). Mathematical modeling is an integral part of the construction of synthetic genetic circuits. Synthetic genetic circuits are typically studied mathematically and computationally before their actual construction in the wet lab. The mathematical model underlying the repressilator is a system of non-linear coupled differential equations. It models three repressor-protein concentrations (lacI, tetR or cI), and their corresponding mRNA concentrations, each of which participate in transcription, translation and degradation reactions. To explore the relevant experimental parameters for the construction of the synthetic system, Elowitz and Leibler performed computer simulations based on this mathematical model. Mathematically, the model was designed to exhibit limit-cycles. Such oscillations are robust to perturbations, and do not damp out. The simulations showed that strong promoters and tight transcriptional repression were conducive for such self-sustained oscillations. This information was utilized in the construction of the repressilator that is an entirely artificial construction engineered to achieve self-sustained oscillations. The three genes of the repressilator do not appear in such a constellation in naturally evolved systems.

**Fig. 1:**
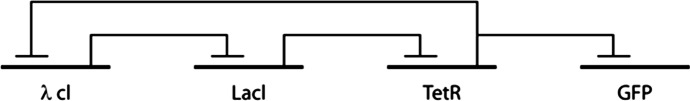
The circuit design of the repressilator. (Wikipedia, 2021)

The synthetic oscillator, the repressilator, was constructed in a wet lab by making use of a plasmid that was introduced into *E. coli* bacteria. Figure 1 shows the basic architecture of the repressilator. The three genes of the repressilator repress each other’s protein production, forming a negative feedback loop. The oscillations on the protein level of the repressilator are made visible by fusing green fluorescent protein (GFP) to the *tetR* gene that enables the study of oscillations through fluorescence microscopy.

Introducing the repressilator into a cell was a tall order. As most of the biochemical parameters remained unknown, it was far from clear whether the repressilator would oscillate as predicted by the mathematical model that functioned as a blueprint for it. But the repressilator started to exhibit oscillations. Writing 16 years after the publication of the repressilator, Elowitz confesses that “[the] appearance of roughly periodic expression of the fluorescent reporter in individual cells was both reassuring and somewhat surprising.” (Gao & Elowitz, 2016, 462).

Although the repressilator oscillated, the oscillations turned out to be noisy, and they varied among the individual cells both in their timing and in amplitude. In order to explore the noisy behavior of the repressilator, the researchers performed computer simulations of a stochastic version of the original mathematical model. The results indicated that the observed fluctuations in the behavior of repressilators were stochastic in nature. This finding posed the questions of how regular oscillations were possible, as well as how they could be synchronized (see Knuuttila & Loettgers, 2021).

**Fig. 2:**
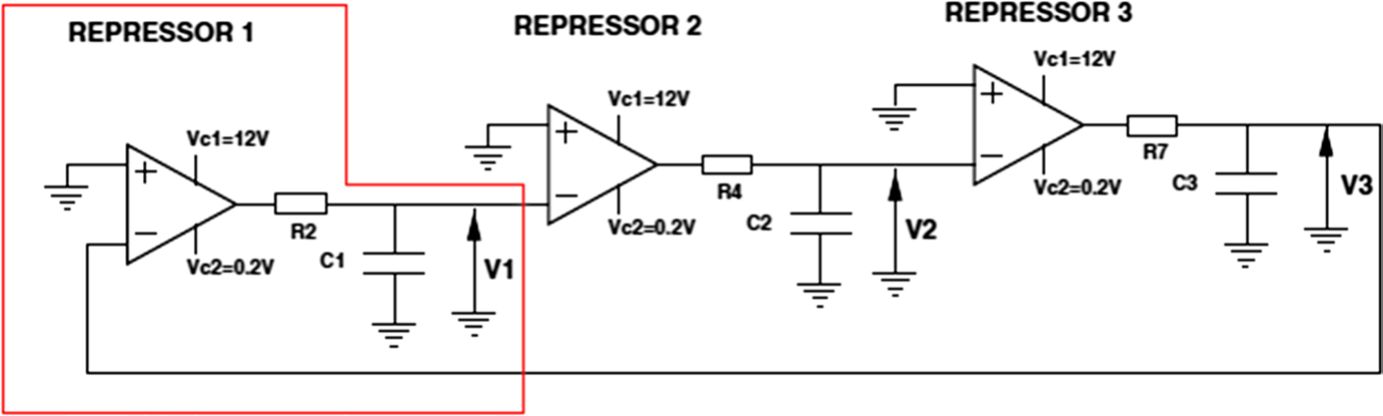
The circuit design of the electronic repressilator (Buldú et al., 2007, 3508)

Interestingly, one way of studying the role of noise and synchronization in genetic circuits was to build an electronic version of the repressilator. The building of the electronic repressilator was motivated by the fact that the wiring diagram that provided the template for the repressilator design is a ring oscillator. It is an electronic circuit composed of an odd number of NOT gates in a ring producing oscillations between two voltages (Fig. 2). Apart from the same basic design, the electronic version of the synthetic oscillator is also “subject to electronic *noise* and *time delays* associated with its operation […].” (Mason et al., 2004, 709, italics added).

The electronic repressilator consists of three elements, each of them modeling a gene, which becomes repressed by the “proteins” produced by its neighbor “gene”. The three basic units are made of RC (R = Resistance C = Capacitor) circuits and operational amplifiers. The voltages $V_1,V_2$ and $V_3$ are analogous to the protein concentrations in the synthetic model. The model exhibits regular oscillations at the three output voltages of the electronic circuit. The behavior of the electronic repressilator shows that in the presence of noise, robust oscillations are still possible. Buldú et al. (2007) also succeeded in getting a population of electronic repressilators to exhibit synchronized oscillations. However, given the problem of relating the electronic and biochemical parameters, they were not able to answer the question of why the colony of synthetic repressilators failed to synchronize.

The research on synthetic oscillators involves sophisticated modal reasoning through its combination of different kinds of modeling. First and foremost, the construction of the synthetic oscillator addresses head-on the question of the biological conceivability of the various feedback designs and mathematical models transferred from physics and engineering to biology. It is difficult to accommodate the aforementioned modeling cycle from the representational perspective: none of the repressilator models – the mathematical, the computational, the synthetic and electronic – aims to represent any naturally evolved circuit. Rather, they are constructed to study the question of whether control at the molecular level could be achieved through a circuit design involving a negative feedback mechanism. Much of the basic science appeal of synthetic biology is based on the reasoning that the truth-maker for a biological possibility would be its realization by engineering means (cf. Contessa, 2010). Moreover, the program of synthetic biology goes further than that: freeing biology from the constraints of evolutionary history. While synthetic biology has unquestionably contributed to the plausibility of engineering approaches in biology, the repressilator, like many other synthetic biology constructs, has so far been only a partial success, remaining in the modal in-between (Knuuttila & Koskinen, 2020).

From an artifactual perspective, the modeling practice of synthetic biology is especially interesting as it testifies to the different enablings and limitations of models embodied in various modes and media. The scientists studying the repressilator were using materially distinct types of models in order to get a better grasp of the phenomenon of intracellular control. The mathematical model and simulation enabled the study of the parameter space conducive to stable oscillations, and the influence of stochasticity. While the mathematical model was relatively easy to configure and consequently handy in studying the network design and the biochemical parameters, it could not answer the question of whether the particular structure studied was realizable in biological systems. The synthetic model was required for that. On the other hand, the biological model, the synthetic oscillator, was both opaque and not easy to manipulate, leading the researchers to study its behavior through constructing an electronic analogue of it. The more easily implementable electronic system allowed for the exploration of the synchronization of oscillators and whether robust oscillations could be possible despite the presence of noise.

## Models for modeling possibilities

Neither the ultra-Keynesian model nor any of the repressilator models in their various incarnations seek to represent an actual real-world target system. The ultra-Keynesian model depicts a fictional system, whose monetary transmission mechanism is not exhibited, or even partially approximated, by actual economies. The repressilator models introduce a minimal biological circuit that is mathematically and computationally studied, and biologically and electronically engineered. According to the representational approach, they would be models of non-actual target systems. However, the claim that they would provide knowledge merely by virtue of representing non-existing targets does not appear to enlighten further vis-à-vis their epistemic functioning.

The artifactual approach provides an alternative perspective on the ultra-Keynesian model and the repressilator models: rather than attributing their epistemic value to a representational relation between a model and a non-existent target system, these models should be appreciated through the questions they are designed to answer. As erotetic devices, these models are targeted towards the phenomena they seek to (re)produce, *internally* representing a system of interpreted dependence relations for engendering the pattern of interest. That the ultra-Keynesian model and the repressilator models are targeted towards phenomena means that they typically aim for dynamical fidelity, i.e. correspondence of the output of the model with the phenomenon of interest. A model that would aim to (externally) represent an actual real-world target system also would need to seek, apart from dynamical fidelity, representational fidelity. Representational fidelity concerns how well the model’s internal structure fits the causal structure of the real-world phenomenon (Weisberg, 2007, 221).

The ultra-Keynesian model seeks to answer the question of whether the empirically established lead and lag patterns between the money supply and nominal income could be brought about by a hypothetical system in which money supply was dependent on nominal income. In order to establish this possibility, Tobin sought to construct a model that was able to replicate Friedman’s empirical findings as closely as possible, with a theoretical structure with opposite causal implications than the model implied by Friedman’s theoretical work and causal claims.

The repressilator models are likewise difficult to accommodate within the representational framework; neither the mathematical model underlying the repressilator, nor the synthetic or electronic systems sought to represent any naturally evolved genetic circuit. The synthetic repressilator is a purposefully designed, simple construct made of well-characterized molecular components. These components are adopted from different contexts of research in view of obtaining robust oscillations. They do not appear in nature in such a constellation. The central question examined with the repressilator is whether feedback systems, such as negative feedback, studied in physics and implemented in various engineering designs, could also be realizable in living cells. Furthermore, if this were the case, the next question would be to what extent biological systems are also organized according to the same general principles as physical sciences? Such a question would not only address the possibilities of constructing novel biological parts and systems, but also the question of whether certain kinds of design principles might be necessary for biological control. Obviously, synthetic biology is far from answering the latter modal question, but the idea of rational reconstruction of novel biological entities and functions seems to presuppose some more general designs principles that would apply to engineering as well as biology.

Modal reasoning comes in, then, through the questions models are designed to answer: the exploration of multiple artifactually rendered systems of interdependencies gives scientists a modal understanding of what is possible, or even necessary. It is important to notice that possibilities studied in this manner address non-actualized entities, systems or processes that might be actualizable or objectively possible. For establishing such objective possibilities, the conceivability of the model is crucial. While generations of economists have been theoretically studying the economic conceivability of the idea of endogenous money supply, synthetic biologists have been able to reach further into the modal realm through the construction of novel biological parts, systems, and functions. The synthetic strategy probes the biological conceivability of different unnatural products, and engineering designs, reaching “beyond the natural” as leading synthetic biologists, Elowitz and Lim (2010) put it. They envision how synthetic biology will expand “biology from a discipline that focuses on natural organisms to one that includes potential organisms” (2010, 889). It seems that such a modal dimension is an integral part of contemporary modeling practice more generally, synthetic biology providing just a particularly dramatic example.

## Footnotes

1 I will use the word ‘mechanism’ in a wider sense than how the new mechanists understand it, i.e. referring to any kind of system able to bring about particular outcomes, without any commitment to decomposition or localization.

2 Since science is a collective activity, one should perhaps rather refer to an inquiry-independent world. Obviously, it is difficult to conceive of those sciences that study the social world and the mind as dealing with mind-independent or even inquiry-independent phenomena.

3 The artifactual approach is loosely related to standard philosophical definitions of artifacts, according to which they are intentionally produced objects whose aim is to accomplish some purpose. In addition to *intentional production*, and the *purpose* artifacts are produced for, a third condition is often added: artifact construction involves *modification of materials* (Preston, 2018).

4 Given that there are different writing systems (e.g. pictographic, logographic, and alphabetic), these different writing systems can in turn be considered as different representational modes, while stone, papyrus, paper, ink, digital computer, or mobiles provide the media of linguistic production (see Kress & van Leeuwen, 2001).

5 The notion of internal representation in the sense I am using it here, does not refer to mental representation.

6 For instance, Frigg (2010) distinguishes between p-representation (where ‘p’ stands for prop) and t-representation (‘t’ for target), see also Salis (2019), and Toon (2012). Such a distinction is also implicit in the distinction between indirect and direct representation (Weisberg, 2007).

7 See Gelfert (2016) for a discussion of exploratory modeling.

8 Obviously, there can also be models of impossible targets.

9 The earlier philosophical studies on the repressilator include Knuuttila and Loettgers (2013a, b), and Weber (2014). These studies concentrate largely on philosophy of biology topics. My intention here is to study the model from an artifactual perspective.
